# A case of eosinophilic esophagitis discovered with positron emission tomography imaging: a case report

**DOI:** 10.1186/1752-1947-7-187

**Published:** 2013-07-15

**Authors:** Bryce D Haslem, Megan I Samuelson, Ron Schey

**Affiliations:** 1 Department of Medicine, Division of Gastroenterology & Hepatology, Univeristy of Iowa, 200 Hawkins Drive, Iowa City, IA 52242, USA; 2 Department of Pathology, University of Iowa Hospitals and Clinics, 200 Hawkins Drive, Iowa City, IA 52242, USA

## Abstract

**Introduction:**

Eosinophilic esophagitis was first reported in 1978, and since then it has been increasingly recognized as one of the major etiologies for dysphagia, food impaction, and food regurgitation. To the best of our knowledge, no case of eosinophilic esophagitis (excluding esophageal eosinophilia not responsive to proton pump inhibitor treatment) has previously been demonstrated on the basis of positron emission tomography imaging.

**Case presentation:**

A 68-year-old Caucasian man presented with dysphagia to solids with recurrent regurgitation and weight loss of 7lb within the preceding 2 months. The patient attributed these symptoms to radiation therapy he had received 1 year earlier for squamous cell cancer of the lung. The patient underwent routine follow-up positron emission tomography imaging, which showed a hypermetabolic lesion in the posterior mediastinum and was increased at the level of the midesophagus.

**Conclusion:**

To the best of our knowledge, this is the first reported case of eosinophilic esophagitis demonstrated by positron emission tomography imaging and confirmed with endoscopic evaluation and biopsies both after positron emission tomography imaging and a trial of proton pump inhibitor therapy. This could have an impact on the diagnostic evaluation of esophageal eosinophilic inflammation as well as eosinophilic infiltration of other gastrointestinal organs.

## Introduction

Eosinophilic esophagitis (EoE) was first reported by Landres *et al.* in 1978, and since then it has been increasingly recognized as one of the major etiologies for dysphagia, food impaction, and food regurgitation [1]. Its prevalence is estimated to be 0.4% in an asymptomatic adult population [2]. Among patients undergoing outpatient upper endoscopy for any indication, the prevalence of EoE varies between 5% to 16% and is highest in patients with dysphagia [3]. The proposed diagnostic criteria for EoE includes more than 15 eosinophils per high-power field (HPF) in the esophageal epithelium. Although the true pathophysiology of EoE remains unknown, the literature has attributed the main etiology of EoE to allergic and genetic causes [4].

## Case presentation

**Figure 1:**
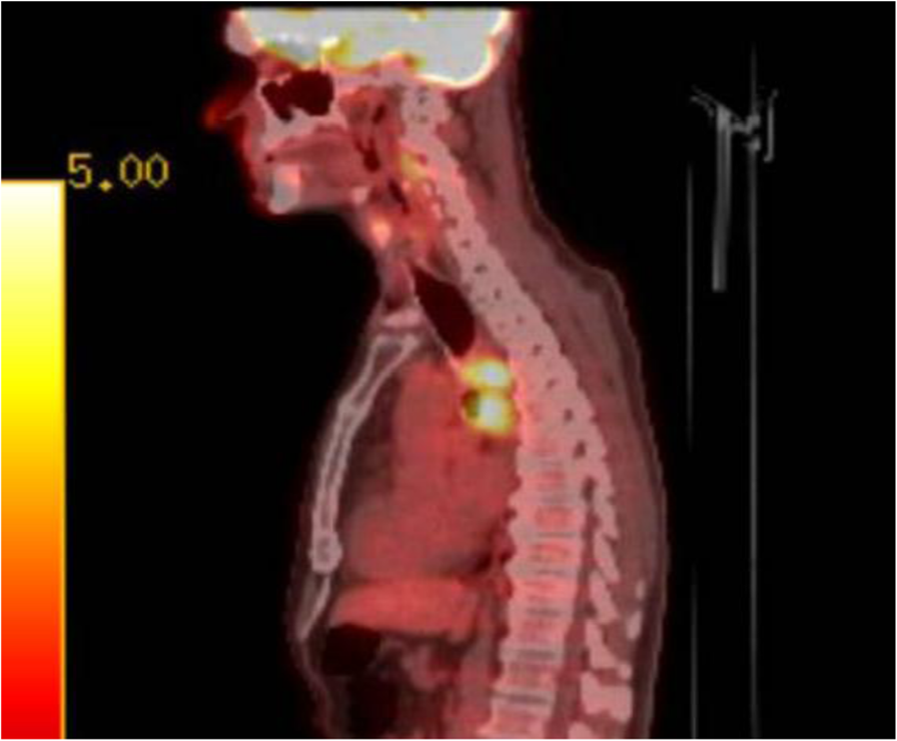
Sagittal positron emission tomography-computed tomography imaging demonstrating a hypermetabolic lesion in the posterior mediastinum with increased levels in the midesophagus.

**Figure 2:**
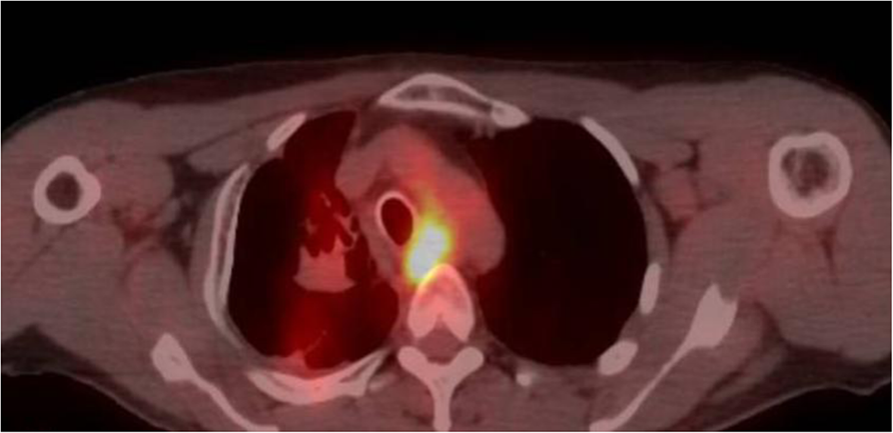
Axial positron emission tomography-computed tomography image demonstrating increased uptake in the midesophagus.

**Figure 3:**
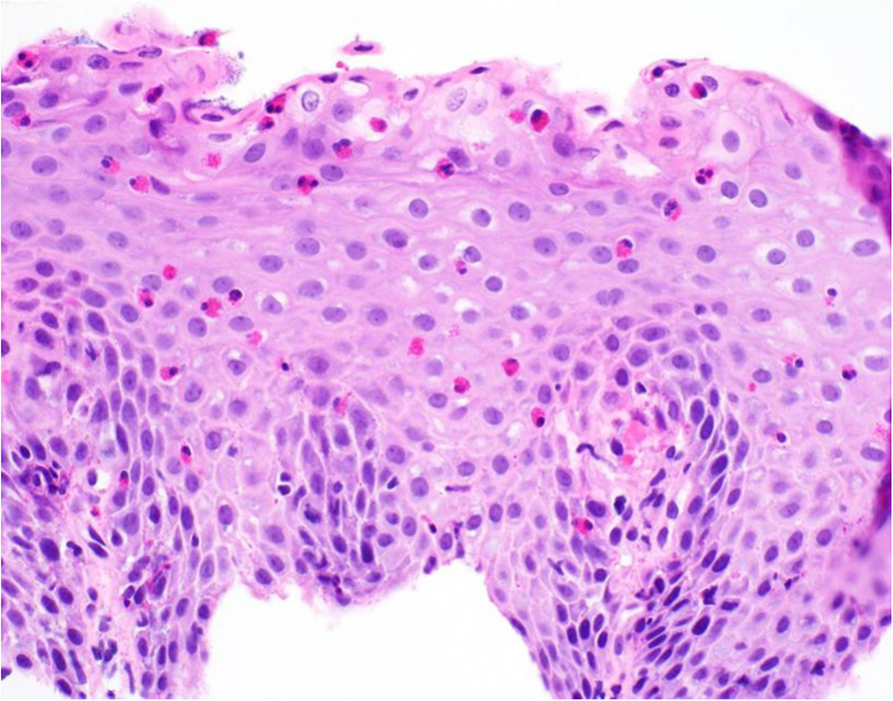
Midesophageal biopsies revealing significant eosinophilia of the midesophagus, with 80 eosinophils per high-power field and associated parakeratosis consistent with eosinophilic esophagitis.

A 68-year-old Caucasian man with a history of squamous cell cancer of the lung in 2011 and a remote history of T4aN2cM0 squamous cell carcinoma of the right oropharynx was referred to our institution because of progressive dysphagia to solids with recurrent regurgitation and loss of 7lb within the previous 2 months. The patient attributed the symptoms to radiation therapy he had received following chemotherapy in late 2011. The patient had done well for more than 1 year and had been followed by primary care and oncology clinicians. Given the worry for malignancy as a cause of his dysphagia, he underwent follow-up positron emission tomography-computed tomography (PET-CT) imaging. This showed a hypermetabolic lesion **(**standardized uptake value 7.6) in the posterior mediastinum increased in the level of midesophagus, as shown in both the axial and sagittal positions (Figures 1 and 2). His physical examination did not reveal any oral thrush or palpable lymphadenopathy. He had no history of allergic disease or atopy. An esophagogastroduodenoscopy (EGD) was performed, which revealed esophageal rings and linear furrowing in the midesophagus without evidence of acid reflux disease. Biopsies from the distal, middle, and proximal esophagus were taken, which revealed significant eosinophilia of the midesophagus with up to 80 eosinophils per HPF and associated parakeratosis consistent with EoE (Figure 3). We elected to perform empiric dilation, and the patient was treated with omeprazole 20mg bid, which led to mild improvement in symptoms but also an episode of food bolus impaction 6 weeks after initiation of treatment. An emergency EGD revealed the same findings, with biopsies showing more than 30 eosinophils per HPF. Swallowed fluticasone 440μg bid was added to the patient’s treatment protocol, which led to a good response. Unfortunately, 8 weeks after treatment the patient died suddenly due to a cerebrovascular accident.

## Conclusions

Although weight loss is not a common presenting symptom of patients with EoE, we believe that, due to weight gain along with alleviation of symptoms after induction of the combined treatment, it would be reasonable to assume that this was the cause of weight loss in our patient.

To the best of our knowledge, there is only one other report of EoE demonstrated by PET imaging and confirmed with endoscopic evaluation and biopsies [5]. However, that report did not comment on proton pump inhibitor therapy (PPI) or other treatment prior to or at the time of follow-up after the PET scan. Therefore, it did not exclude PPI-responsive esophageal eosinophilia (PPI-REE).

In our patient, there was intense eosinophilia of the affected area of the midesophagus, with up to 80 eosinophils per HPF with repeat biopsies after 6wk of treatment showing more than 30 eosinophils per HPF. His history of long-term symptoms of dysphagia, which became more progressive, are typical of patients presenting with EoE. Previous reports have described CT findings of thickening of the esophagus related to EoE [6]. It is likely that other inflammatory conditions of the esophagus would show similar results and could include infectious esophagitis, reflux esophagitis, malignancy, and others. Given the cost associated with PET-CT imaging and the broad differential of positive findings, we do not suggest that this modality has a primary role in diagnosing EoE at this time. However, it can potentially provide another diagnostic modality besides upper endoscopy. It may also provide information which could distinguish between PPI-REE and EoE, which could affect treatment decisions. More studies need to be done in this area, but in our patient the PET imaging demonstrated intense inflammation caused by eosinophilic infiltration which did not respond to PPI treatment.

## Consent

Written informed consent was obtained from the patient’s spouse for publication of this case report and accompanying images. A copy of the written consent is available for review by the Editor-in-Chief of this journal.

## Competing interests

The authors declare that they have no competing interests.

## Authors’ contributions

BH collected data and was a major contributor in writing the case report. MS was the pathology consult and provided critical revision of the case report. RS collected and interpreted the findings, was the endoscopist and investigator, obtained informed consent, and was a major contributor in writing the case report. All authors read and approved the final case report.

## References

[B1] Landres RT Kuster GG Strum WB Eosinophilic esophagitis in a patient with vigorous achalasia Gastroenterology 1978 74 1298 1301 648822

[B2] Straumann A Simon HU Eosinophilic esophagitis: escalating epidemiology? J Allergy Clin Immunol 2005 115 418 419 10.1016/j.jaci.2004.11.006 15696105

[B3] Veerappan GR Perry JL Duncan TJ Baker TP Maydonovitch C Lake JM Wong RK Osgard EM Prevalence of eosinophilic esophagitis in an adult population undergoing upper endoscopy: a prospective study Clin Gastroenterol Hepatol 2009 7 420 426 426.e1–e2 10.1016/j.cgh.2008.10.009 19162236

[B4] Blanchard C Wang N Rothenberg ME Eosinophilic esophagitis: pathogenesis, genetics, and therapy J Allergy Clin Immunol 2006 118 1054 1059 10.1016/j.jaci.2006.07.038 17088129

[B5] Dong A Wang Y Zuo C FDG PET/CT in eosinophilic esophagitis Clin Nucl Med 2013 38 e118 e124 10.1097/RLU.0b013e318270868a 23510886

[B6] Horiki N Maruyama M Fujita Y Yonekura T [A case of idiopathic eosinophilic esophagitis with CT findings showing marked thickening of the esophageal wall] [in Japanese] Nihon Shokakibyo Gakkai Zasshi 1998 95 769 776 9721518

